# Extended classifier system with continuous real-coded variables for feature extraction of instantaneous pulse-rate variability and respiration of individuals with gaming disorder

**DOI:** 10.1186/s12938-021-00930-3

**Published:** 2021-09-23

**Authors:** Hung-Ming Chi, Tzu-Chien Hsiao

**Affiliations:** 1 Department of Computer Science, College of Computer Science, National Yang Ming Chiao Tung University, 1001 University Road, Hsinchu, Taiwan, ROC; 2 Institute of Biomedical Engineering, College of Electrical and Computer Engineering, National Yang Ming Chiao Tung University, 1001 University Road, Hsinchu, Taiwan, ROC

**Keywords:** Empirical mode decomposition, Gaming disorder, Machine learning system, Instantaneous pulse rate variability

## Abstract

**Background:**

Individuals with gaming disorder (GD) exhibit autonomic nervous system responses that indicate dysfunctional emotion regulation. Pulse rate variability (PRV) is a valuable biomarker for investigating the autonomic function of patients with mental disorders. Because individuals with GD dynamically regulate emotions during gaming, the PRV response relating to GD is not well understood. To investigate the dynamic PRV responses of individuals with GD, this study proposed the indexes of instantaneous PRV (iPRV) and instantaneous respiratory frequency (IF_resp_) of arterial blood pressure signals using empirical mode decomposition and normalized direct-quadrature algorithms. iPRV consists of low-frequency (LF), high-frequency (HF), and very high-frequency (VHF) bands. Moreover, a novel method of extended classifier system with continuous real-coded variables (XCSR) was used to detect GD and extract GD-related iPRV features using iPRV and IF_resp_ as input data.

**Results:**

A total of 32 college students without depressive and anxiety symptoms or cardiovascular diseases were recruited in this study. Participants were grouped into the high-risk GD and low-risk GD using both Chen Internet Addiction Scale and Internet Gaming Disorder Questionnaire. Their arterial blood pressures signals were measured while they watched gameplay videos with negative or positive emotional stimuli. Seven participants with high-risk GD exhibited significantly increased normalized VHF (nVHF) PRV and IF_resp_ readings and significantly decreased normalized LF (nLF) PRV readings and LF/HF PRV ratios (from baseline) during negative or positive gameplay videos stimuli. These participants also exhibited higher nVHF PRV and lower nLF PRV readings and LF/HF PRV ratios when they experienced negative gameplay video stimuli relative to 17 participants with low-risk GD. The classification accuracy of the XCSR reached 90% for both negative and positive video stimuli, and nVHF PRV was most frequently used to detect GD risk.

**Conclusions:**

iPRV and IF_resp_ can be used to detect GD and analyze the autonomic mechanism of individuals with GD.

## Background

With the rapid development of the gaming industry, students can play online games using desktop computers, laptops, smartphones, and other mobile devices. For the age distribution of gamers, 35% of gamers worldwide were between the ages of 21 and 35 years in 2017, and 28% of gamers were under the age of 18 in the USA in 2018 [1]. Some gamers, however, exhibit loss of control and excessive and persistent gaming behavior that may limit their educational opportunities, relationship with their family and friends, and psychological and physical development [2]. Students can access the Internet easily which makes them be a potential GD risk [3]. In 2013, Internet gaming disorder (GD) was provisionally listed in Section III of the Diagnostic and Statistical Manual of Mental Disorders, 5th Edition [2]. In 2019, GD was officially classified by the 72nd World Health Assembly as a type of mental, behavioral, or neurodevelopment disorder, and it was added to the 11th revision of the International Classification of Diseases [4]. GD has become a major problem worldwide.

In the neurobiological mechanism of addiction, emotion plays a key role in decision-making relating to substance use and activities involving drugs, gambling, and Internet use [5, 6]. Some researchers have suggested that individuals with GD are at a heightened risk of dysfunctional emotional regulation, which is characterized by neuroticism, aggression, poor self-control, anxiety, and depression [7–9]. Emotional regulation affects the cardiac autonomic function of individuals with GD [10–13]. Heart rate variability (HRV), which is the variation in the time interval between consecutive heartbeats, has been used to investigate the cardiac autonomic function of individuals with GD. In a study in which participants were subjected to familial stresses lasting 5 min, the GD risk was discovered to be negatively related to the respiratory sinus arrhythmia, which is a measure of HRV [11]. Gamers with GD exhibited a decrease in the natural logarithm of high-frequency (HF) HRV when they focused on gaming for 5 min [12]. Compared with the non-GD control group, a GD group exhibited a lower logarithm of low-frequency (LF) and HF HRV; they also experienced greater negative affectivity and inhibition of emotional expression [13]. Individuals with GD exhibited decreased HF HRV and increased LF/HF HRV ratios when playing online games for 5 min [10]. Although the HRV responses of gamers with GD have been investigated, the neurobiological mechanism is still unclear.

Moreover, HRV analysis has three limitations. First, HRV analysis mainly involves using fast Fourier transform, which cannot analyze nonlinear and nonstationary signals because it is a linear stationary mathematic framework. Second, HRV analysis uses interpolation to produce a tachogram, which affects the time resolution of the power spectrum. Third, HRV is primarily evaluated using 5-min electrocardiogram readings, and few studies have investigated instantaneous changes in HRV. Researchers have proposed the use of empirical mode decomposition (EMD), which is an adaptive filter algorithm that can decompose nonlinear or nonstationary signals [14]. The normalized direct-quadrature (NDQ) method is a time–frequency analysis method that can calculate the instantaneous frequency of continuously nonlinear signals [15]. The EMD and NDQ methods can be applied to overcome the limitations of HRV analysis.

An EMD-based method can decompose an arterial blood pressure (ABP) signal into pulse beat- or respiratory-related oscillation components [16–18]. ABP signal, which reveals information on changes in the pulse-to-pulse interval of peripheral tissue, is related to heartbeat. Pulse rate variability (PRV) is the variation of time series intervals between the pulse-to-pulse interval of ABP signals, and it provides more information on cardiac and vascular autonomic responses than HRV [19]. The relationship between pulse beat and respiration can also be used to investigate cardiorespiratory adjustments [16, 18]. PRV has been used to evaluate the autonomic function of individuals with mental illness [20, 21]. The pulse beat waveform can be transformed into instantaneous PRV (iPRV) using time–frequency analysis methods, and iPRV responses provide information on not only parasympathetic nervous system (PNS) and sympathetic nervous system (SNS) activities, but also peripheral circulation [17, 18]. Psychologists can use iPRV to explore instantaneously autonomic and peripheral regulation and thus understand the neurobiological mechanisms underlying GD-related emotions.

Some researchers have also suggested using psychophysiological features as data sets and inputting them into machine learning systems designed to detect addiction [22–24]. However, these researchers may not know the key psychophysiological features that can be used as indexes. An extended classifier system with continuous real-coded variables (XCSR) is a rule-based learning classifier system [24, 25], and the rule is linear combiners [26]. The XCSR can interact with the environment to solve real-value problems; moreover, researchers can use an XCSR to observe the weight of each input feature [24, 25]. The hypotheses of this study are that iPRV of ABP signals can be evaluated using EMD-based and NDQ methods, and an XCSR can detect GD risk and extract GD-related psychophysiological features. Therefore, this study aimed to (1) calculate the iPRV of gamers with high-risk GD (HGD) using a combination of EMD-based and NDQ methods and to (2) assess the classification performance of an XCSR and the selection rate of each iPRV feature.

## Results

17 participants (13 men) and 7 participants (6 men) were classified into low-risk GD (LGD) and HGD using both Chen Internet Addiction Scale (CIAS) and Internet Gaming Disorder Questionnaire (IGDQ), respectively. CIAS and IGDQ were used to assess Internet addiction [27] and GD [28], respectively. Table 1 presents the demographic information and questionnaire scores of participants with LGD and HGD. The Internet Gaming Disorder Questionnaire (IGDQ) and Chen Internet Addiction Scale (CIAS) scores of participants with HGD were both significantly higher than those of participants with LGD (Mann–Whitney *U* test, *p* < 0.001). No significant difference was observed between the two groups for age and Self-Assessment Manikin (SAM, assessment of emotional valence and arousal [29]) and Discrete Emotions Questionnaire (DEQ, assessment of intensities of happiness, surprise, anger, disgust, fear, and sadness) scores (Mann–Whitney *U* test, *p* > 0.05). After LGD and HGD groups watched a League of Legends gameplay video of 6 min (also called Stimulus 1), the emotional valence scores were higher than 5, and happiness and surprise scores were higher than anger, sadness, disgust, and fear scores. Stimulus 1 was approaching the elicitation of positive emotions. After two groups watched a Resident Evil gameplay video of 6 min (also called Stimulus 2), the emotional valence scores were lower than 5, and surprise, disgust, and fear scores were higher than happiness, anger, and sadness scores. The Stimulus 2 was approaching the elicitation of negative emotions.

**Table 1:** Demographic information and questionnaire scores of the LGD and HGD groups

Characteristic		LGD (*n* = 17)	HGD (*n* = 7)	*p*-value
Gender (men, women)		13, 4	6, 1	0.62
Age		23 ± 3	22 ± 1	0.58
IGDQ		0.94 ± 1.20	6.57 ± 1.51	< 0.001
CIAS		57.24 ± 4.35	78.71 ± 10.23	< 0.001
Stimulus 1	SAM_valence	6.59 ± 1.33	7.29 ± 0.76	0.21
	SAM_arousal	4.18 ± 2.35	5.71 ± 2.50	0.14
	DEQ_happiness	3.82 ± 1.74	5.86 ± 2.61	0.09
	DEQ_surprise	2.35 ± 1.77	3.86 ± 3.08	0.35
	DEQ_anger	1.41 ± 1.23	2.43 ± 1.81	0.11
	DEQ_sadness	1.29 ± 0.99	2.29 ± 1.98	0.23
	DEQ_disgust	1.29 ± 1.21	1.14 ± 0.38	0.80
	DEQ_fear	1.00 ± 0.00	1.43 ± 1.13	0.62
Stimulus 2	SAM_valence	4.65 ± 1.22	5.14 ± 1.77	0.58
	SAM_arousal	6.59 ± 1.77	6.29 ± 1.98	0.70
	DEQ_happiness	2.82 ± 1.70	3.14 ± 2.04	0.80
	DEQ_surprise	5.12 ± 1.45	5.86 ± 2.54	0.49
	DEQ_anger	1.29 ± 0.85	2.00 ± 1.00	0.13
	DEQ_sadness	2.00 ± 1.46	1.57 ± 0.79	0.85
	DEQ_disgust	5.65 ± 2.26	4.86 ± 3.34	0.62
	DEQ_fear	5.18 ± 2.24	4.00 ± 2.31	0.26

CIAS: Chen Internet Addiction Scale; DEQ: Discrete Emotions Questionnaire; HGD: high-risk gaming disorder; IGDQ: Internet Gaming Disorder Questionnaire; LGD: low-risk gaming disorder; SAM: Self-Assessment Manikin; *p* value for Mann–Whitney *U* test comparing LGD and HGD

Table 2 summarizes the iPRV and instantaneous respiratory frequency (IF_resp_) results of the two groups for the two trials. Trial 1 contains gazing at a gray picture of 6 min (Baseline 1) and Stimuli 1. Trial 2 contained gazing at a gray picture of 6 min (Baseline 2) and Stimuli 2. The iPRV and IF_resp_ of ABP signals were calculated using complementary-ensemble EMD (CEEMD) and NDQ methods. The iPRV includes LF, HF, very high-frequency (VHF), LF/HF, nLF (LF/(LF + HF + VHF)), nHF (HF/(LF + HF + VHF)), and nVHF (VHF/(LF + HF + VHF)). The results in Trial 1 indicated that participants with HGD exhibited higher VHF PRV, nVHF PRV, and IF_resp_ and lower LF/HF PRV ratios and nLF PRV during Stimulus 1 relative to Baseline 1 (factorial ANOVA, *p* < 0.01). Participants with LGD exhibited higher nVHF PRV and IF_resp_ and a lower LF/HF PRV ratio during Stimulus 1 relative to Baseline 1 (factorial ANOVA, *p* < 0.01). Compared with the LGD group, the HGD group exhibited higher LF PRV and lower IF_resp_ at Baseline 1 and higher LF, HF, and VHF PRV during Stimulus 1 (factorial ANOVA, *p* < 0.01). The results in Trial 2 showed that participants with HGD exhibited higher nVHF PRV and IF_resp_, but a lower LF PRV, LF/HF PRV ratio, and nLF PRV during Stimulus 2 relative to Baseline 2 (factorial ANOVA, *p* < 0.01). Compared with the LGD group, the HGD group exhibited higher LF PRV and lower IF_resp_ at Baseline 2 and higher HF, VHF, and nVHF PRV and a lower LF/HF PRV ratio and nLF PRV during Stimulus 2 (factorial ANOVA, *p* < 0.01).

**Table 2:** Mean ± standard deviation of the iPRV and IF_resp_ of participants with LGD and HGD

feature	LGD			HGD	
Trial 1	Baseline 1	Stimulus 1		Baseline 1	Stimulus 1
LF × 10^3^ (ms^2^)	4.77 ± 3.00	4.61 ± 4.30		9.64 ± 8.82^§^	9.33 ± 12.62^§^
HF × 10^3^ (ms^2^)	4.36 ± 4.85	5.30 ± 8.46		4.94 ± 3.72	10.22 ± 13.58^§^
VHF × 10^3^ (ms^2^)	7.19 ± 9.08	9.30 ± 14.19		10.34 ± 7.60	27.61 ± 41.37^*§^
LF/HF ratio	2.22 ± 2.16	1.55 ± 1.03^*^		2.60 ± 2.11	1.37 ± 0.56^*^
nLF (%)	37.31 ± 17.44	32.02 ± 15.31		37.11 ± 14.35	27.70 ± 8.45^*^
nHF (%)	23.87 ± 8.83	23.89 ± 5.96		21.26 ± 9.76	23.08 ± 7.97
nVHF (%)	38.82 ± 11.53	44.09 ± 11.93^*^		41.62 ± 7.76	49.21 ± 10.04^*^
IF_resp_	0.19 ± 0.08	0.23 ± 0.10^*^		0.14 ± 0.07^§^	0.21 ± 0.10^*^
Trial 2	baseline_2	stimuli_2		baseline_2	stimuli_2
LF × 10^3^ (ms^2^)	6.19 ± 6.17	4.73 ± 4.04		12.69 ± 13.81^§^	6.68 ± 5.43^*^
HF × 10^3^ (ms^2^)	5.50 ± 7.31	4.61 ± 4.27		7.59 ± 7.92	9.47 ± 11.20^§^
VHF × 10^3^ (ms^2^)	10.11 ± 14.40	8.52 ± 8.50		16.36 ± 15.01	18.05 ± 18.17^§^
LF/HF ratio	1.69 ± 1.40	1.34 ± 0.77		2.15 ± 1.10	1.03 ± 0.39^*§^
nLF (%)	32.44 ± 15.44	29.79 ± 13.97		36.64 ± 10.41	23.05 ± 7.48^*§^
nHF (%)	23.90 ± 6.90	24.37 ± 6.34		20.78 ± 7.93	23.91 ± 5.98
nVHF (%)	43.66 ± 11.32	45.84 ± 12.96		42.58 ± 7.00	53.05 ± 9.07^*§^
IF_resp_	0.22 ± 0.08	0.24 ± 0.07		0.16 ± 0.07^§^	0.22 ± 0.10^*^

*p < 0.01 for factorial ANOVA comparing baseline and stimulus

^§^*p* < 0.01 for factorial ANOVA comparing LGD and HGD groups

**Fig. 1:**
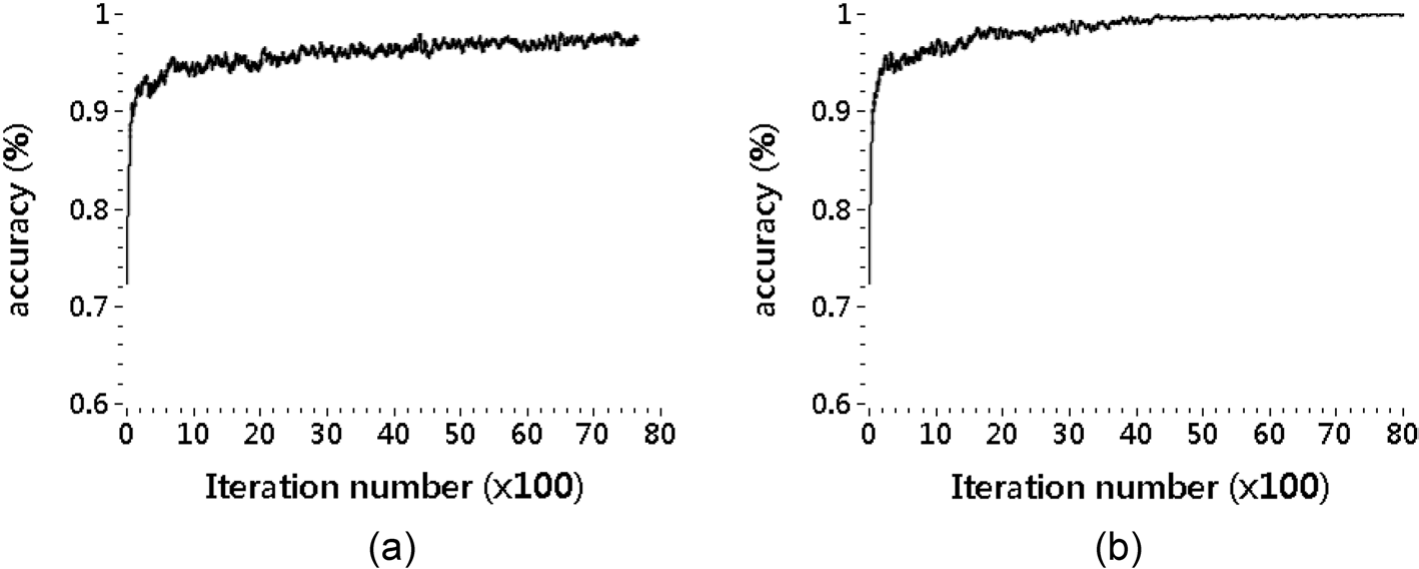
Classification accuracies of the XCSR for Stimuli 1 (**a**) and 2 (**b**)

**Fig. 2:**
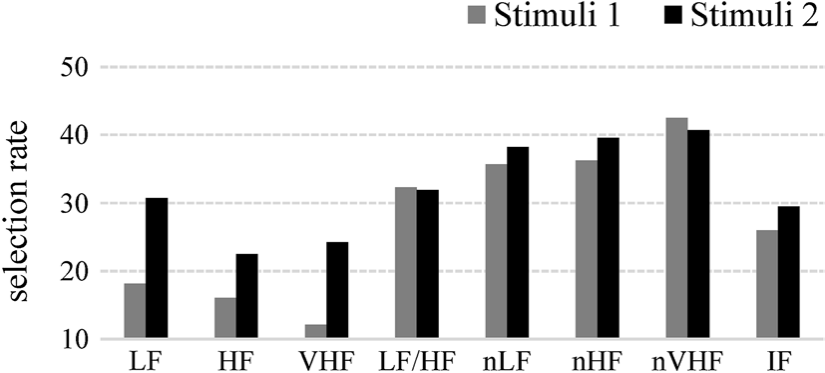
Selection rates of iPRV and IF_resp_ for both stimuli

The classification accuracy of the XCSR was examined using the moving average per 50 exploitations. Figure 1 presents the classification accuracies of the XCSR of an average of 30 repetitions, which were determined with iteration number 8,400 using iPRV and IF_resp_ as input features in Stimuli 1 and 2. The results exhibit that the classification accuracies for Stimulus 1 and 2 were above 90%. If the conditions of classifiers in the XCSR did not match the input features (LF PRV, HF PRV, VHF PRV, LF/HF PRV ratio, nLF PRV, nHF PRV, nVHF PRV, and average of IF_resp_), these conditions were ignored. The selection rate of each feature was determined by dividing the total number of the collected classifiers by the number of conditions that were not ignored. Figure 2 presents the selection rates of the features of IF_resp_ and LF, HF, VHF, LF/HF ratio, nLF, nHF, and nVHF PRV during Stimulus 1 and 2. For the two stimuli, the feature evaluated for nVHF PRV exhibited the highest selection frequency.

## Discussion

This study evaluated the instantaneous pulse rate and IF_resp_ of ABP signals through the combined application of CEEMD and NDQ methods. The power spectrum of instantaneous pulse rate (i.e., iPRV) contained LF, HF, and VHF values. iPRV was examined to determine the autonomic function and peripheral circulation of participants with HGD and LGD when they watched League of Legends (positive emotional stimuli) and Resident Evil (negative emotional stimuli) gameplay videos. Furthermore, the XCSR was used to assess the classification accuracy for GD and investigate the selection rates of iPRV features (LF, HF, VHF, LF/HF ratio, nLF, nHF, and nVHF) and IF_resp_.

In the present study, we observed a statistically significant increase in nVHF PRV, and IF_resp_ and decrease in nLF PRV and LF/HF PRV ratios among participants with HGD with respect to the baseline versus positive and negative emotional stimuli results. The participants with LGD also exhibited statistically significantly increased nVHF PRV and IF_resp_ and a decreased LF/HF PRV ratio with respect to baseline versus positive emotional stimuli results. Our findings, however, do not fully correspond to those of previous studies. Relevant studies have reported the following: posttraumatic stress disorder symptom is negatively associated with HF PRV during rest [20], gamers with GD tend to exhibit an increased LF/HF HRV ratio [10] and respiratory rates [22] and decreased natural logarithm of LF HRV [12] when playing online games, gamers with GD exhibit increased respiratory rates when watching gameplay videos [30], and individuals exhibit increased total peripheral resistance when performing negative memory recall [31] and experiencing positive video stimuli [32]. This inconsistency can be attributed to the varying respiratory and cardiovascular responses triggered by varying emotional stimuli, particularly in the HGD group. Furthermore, respiration influences HR and BP through the vagus nerve, which modulates the balance between SNS and PNS [33]. When individuals’ breathing rates are between 0.15 and 0.20 Hz or 0.15 and 0.25 Hz, they exhibit a decreased LF HRV and LF/HF HRV ratio [34]. Compared with individuals who are spontaneously breathing, individuals with a 0.1 Hz breathing rate exhibit increased LF iPRV, and those with a 0.5 Hz breathing rate exhibit increased VHF iPRV [18]. Our findings suggest that the positive and negative game-related cues may increase the respiratory rates of gamers with HGD and that these respiratory rates may affect their autonomic function and peripheral vascular regulation. How respiration influences the cardiovascular responses of gamers with HGD is a topic requiring further investigation.

Compared with the participants with LGD, those with HGD exhibited statistically significantly higher VHF, HF, and LF PRV when experiencing positive emotional stimuli and higher nVHF, VHF, and HF PRV as well as a statistically significant lower nLF PRV and LF/HF PRV ratio when experiencing negative emotional stimuli. These results contradict those of previous studies, which reported that gamers with GD exhibit lower HF HRV [10] and lower logarithm of LF and HF HRV [13] when they are playing games. This inconsistency may be explained by the psychophysiological differences between participants and the fact that VHF HRV was not considered. The replacement of HRV with PRV is still a debated topic [19]. Nonetheless, our results may be explained by the roles of external and internal stimuli in encouraging cerebral cortices to regulate emotions, cognition, or attention. Neurotransmitters output from these cerebral cortices via the preganglionic nerves of the SNS and PNS, which, in turn, influences cardiovascular responses [35]. Relative to gamers without GD, gamers with GD exhibit higher blood flow in the cerebral cortices when experiencing negative picture stimuli, which suggests that gamers with GD focus more on coping with negative emotions than positive emotions [36]. Our findings suggest that gamers with HGD have dysfunctional emotional regulation, particuarly with respect to negative emotions. This dysfunction may have led to differences in the autonomic function and peripheral circulation of the HGD and LGD groups.

The classification accuracy of the XCSR when iPRV and IF_resp_ were used as features was more than 90% for both positive and negative video stimuli. Researchers have recommended biosignals (including eye blinking, skin conductance, and heart and respiratory rates) with the use of support vector machines to classify the cravings of HGD groups during gaming activities [22]. Respiratory muscle contraction and respiratory wall movement frequency were also used in an XCSR to determine the GD risk [24]. Therefore, we infer that iPRV and IF_resp_ can be used as psychophysiological indexes to detect LGD and HGD. Our results also indicated that nVHF PRV was most frequently selected to detect GD risk; however, its selection rate was less than 50%. This can be explained by the small sample size and the parameter setup of the XCSR, which may have affected the generation of classifiers in [P]. Despite the limitations of sample size, some studies have used VHF PRV readings to investigate the autonomic nervous function of patients with chronic heart failure [37] and cardiac autonomic neuropathy [38]. VHF readings can be used as an index for evaluating GD risk. More data are required to clarify the physiological interpretation of VHF PRV readings with respect to gamers with HGD.

This study had several limitations. First, the majority of gamers are male; only four female gamers were enrolled in this study; therefore, our findings may contain gender bias. Second, different cue stimuli (such as the use of different game types, technological devices, and passive and active stimuli) may trigger different iPRV responses. Third, some participants were not familiar with the League of Legends or Resident Evil games, which is a factor that might have also introduced bias into the results. Fourth, the setup of parameters for the CEEMD method might have influenced the decomposition of ABP signals. Fifth, we did not investigate the differences between iPRV and HRV; these differences might have provided more information on the cardiovascular responses of participants with HGD. Sixth, we didn’t compare our approach with other studies’ methods. Further research will investigate this comparison.

## Conclusion

The present study evaluated the iPRV and IF_resp_ of participants with LGD and HGD. Furthermore, the frequency bands of iPRV and IF_resp_ were used as data sets and input into an XCSR to assess the classification power for GD risk. The XCSR was also used to evaluate the selection rate of each feature. The results indicated that relative to their baseline readings, participants with HGD exhibited higher nVHF PRV and IF_resp_ and lower nLF PRV and LF/HF PRV ratios when watching positive or negative gameplay videos. Compared with participants with LGD, those with HGD also exhibited higher nVHF PRV and lower nLF PRV and LF/HF PRV ratios when experiencing negative stimuli. The classification accuracy was above 90% for both positive and negative video stimuli, and nVHF PRV was the most frequently selected GD-related psychophysiological feature. We infer that games aroused emotional responses in participants with HGD, whose respiratory frequency increased to enable both autonomic function and peripheral circulation adjustments. This study has two contributions, iPRV and IF_resp_ evaluations using a combination of EMD-based and NDQ methods, and detection of IA risk and extraction of GD-related iPRV features using XCSR method, respectively. iPRV may be useful as a feature for exploring the dynamically psychophysiological regulation of gamers with HGD, particularly for nVHF PRV responses. The XCSR model may provide researchers to extract the psychophysiological properties of GD. Future studies could investigate the effect of respiration of gamers with HGD on their cardiovascular responses when they play online games for long durations.

## Method

### Participants and data collection

In total, 32 college students (28 male and 4 female students aged between 20 and 33 years) without depressive and anxiety symptoms or cardiovascular diseases were recruited at National Chiao Tung University, Taiwan. All participants signed an informed consent form and were evaluated for risk of Internet addiction and Internet GD using the CIAS [27] and IGDQ [28], respectively. The CIAS is a 26-item questionnaire answered on a 4-point Likert-type scale. It evaluates respondents’ experience of Internet use. The IGDQ is a 9-item questionnaire that uses dichotomous items to assess respondents’ experience of playing online games. Participants were categorized as having HGD if they had CIAS and IGDQ scores of > 64 and > 5, respectively. The SAM, a commonly used questionnaire, was adopted to evaluate emotional valence and arousal [29]. The DEQ was used to assess discrete emotions such as happiness, surprise, anger, sadness, disgust, and fear. Both the SAM and DEQ use answered on a 9-point Likert-type scale ranging from 1 to 9. The emotional valence score is 5 that represented neutral emotion. The emotional valence scores are higher than 5 that represented positive emotions, and the emotional valence scores are lower than 5 that represented negative emotions. The emotional arousal and DEQ expressed emotional intensity from 1 (low) to 9 (high). League of Legends and Resident Evil gameplay videos were used as passive stimuli to elicit the desire to play online games. League of Legends is a popular game worldwide [39]; Resident Evil has been classified as a violent game that is positively associated with aggression [40].

**Fig. 3:**
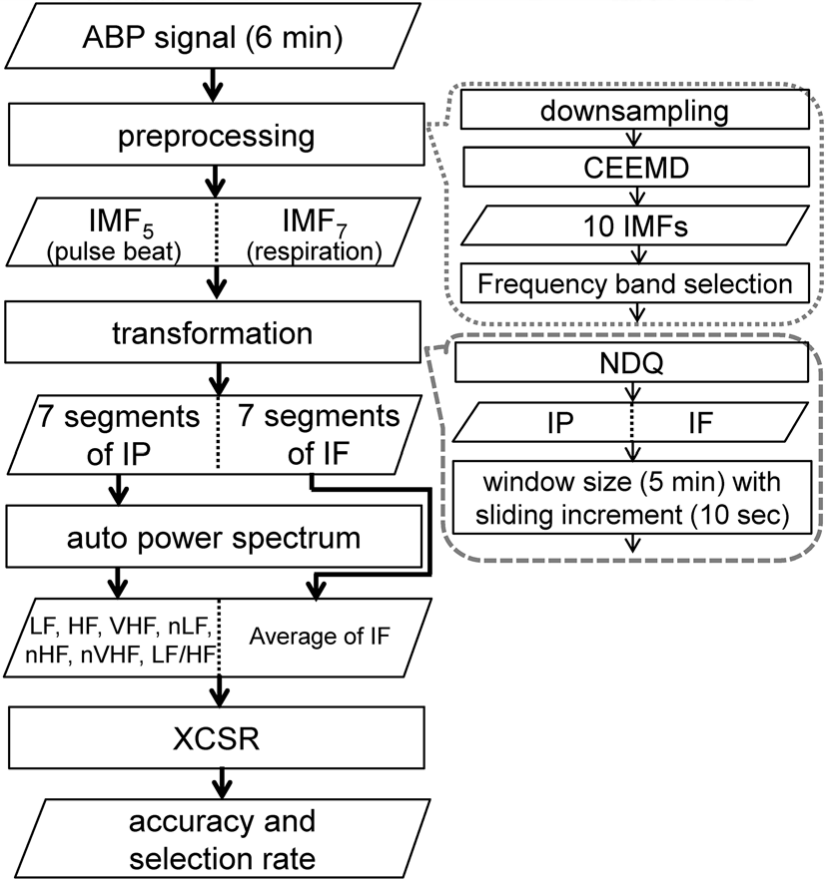
Processing procedure for ABP signals

The experimental procedure was as follows. A participant first executed the isovolume maneuver [41] (i.e., abdominal breathing exercises) for 10 min to attain a state of calmness. Next, the participant was asked to complete Trials 1 and 2. For each trial, the participant gazed at a gray picture for 6 min to achieve relaxed psychophysiological responses (which were used as the baseline), viewed a gameplay video (which was used as an emotional stimulus) for 6 min, and then filled out the DEQ and SAM questionnaire (which were used as emotional assessment tools) under no time constraints. During the stimulus phase, the participant watched a League of Legends gameplay video (Stimulus 1) for Trial 1 and a Resident Evil gameplay video (Stimulus 2) for Trial 2. The order in which the two trials were conducted was randomized. During the experiment, ABP signals were measured using a noninvasive blood pressure system (CNAP Monitor 500, CNSystems Medizintechnik, Graz, Austria), which was acquired using DAQCard (USB 6218, NI, Austin, TX, USA) with a sampling rate of 1 kHz. The signal analysis procedure is presented in Fig. 3. The ABP signals recorded during the 6-min baseline and stimulus phases were analyzed. Data collection and analysis were performed using LabVIEW (version 2020, NI, Austin, TX, USA).

### Signal decomposition through complementary-ensemble EMD

The analysis process for EMD is as follows [14]

- 1) Detect the peak and valley points of signal *x*(*t*) to draw local maxima and minima, respectively.
- 2) Generate the upper envelope *u*_0_(*t*) and lower envelope *l*_0_(*t*) using a cubic spline and calculate the mean envelope *m*_0_(*t*) using the following equation (1):
  1 $${m_0}(t) = \frac{{{u_0}(t) + {l_0}(t)}}{2}$$
- 3) Subtract *m*_0_(*t*) from *x*(*t*) to compute the local oscillation *h*_0_(*t*) using the following equation (2):
  2 $${h_0}(t) = x(t) - {m_0}(t)$$
- 4) Repeat Steps 1 to 3 until *m*_*j*_(*t*) approaches zero, at which point *h*_*j*_(*t*) is considered the first intrinsic mode function (IMF) and referred to as IMF_1_(*t*).
- 5) Subtract IMF_1_(*t*) from *x*(*t*) to compute the first residue *r*_1_(*t*), which is then input into step 1.
- 6) Iterate steps 1 to 5. After *n* iterations, the signal *x*(*t*) is decomposed into *n* IMFs and expressed using the following equation (3):
  3 $$x(t) = \sum\limits_{i = 1}^n {{\text{IM}}{{\text{F}}_i}{(}t{) + }{r_n}{(}t{)}}$$

To overcome the mode mixing shortcomings of EMD, CEEMD was proposed; the decomposition procedure for CEEMD is as follows [42]:

- 1) Add the positive and negative Gaussian white noises to signal *x*(*t*) to establish, respectively, the two new signals *x*^+^(*t*) and *x*^-^(*t*).
- 2) Decompose *x*^+^(*t*) and *x*^-^(*t*) using EMD, which expressed the following equation (4) and (5):
  4 $${x^+ }(t) = \sum\limits_{i = 0}^n {{\text{IMF}}_i^+ (t) + r_n^+ (t)}$$
  5 $${x^- }(t) = \sum\limits_{i = 0}^n {{\text{IMF}}_i^- (t) + r_n^- (t)}$$
- 3) Repeat Steps 1–2 for *N* times to construct two ensembles of IMFs for *x*^+^(*t*) and *x*^-^(*t*).
- 4) Compute the final IMF using the following equation (6):
  6 $${\text{IM}}{{\text{F}}_i}(t) = \frac{1}{2N}\sum\limits_{k = 1}^N {[{\text{IMF}}_{ki}^+ (t) + {\text{IMF}}_{ki}^- (t)]}$$

**Fig. 4:**
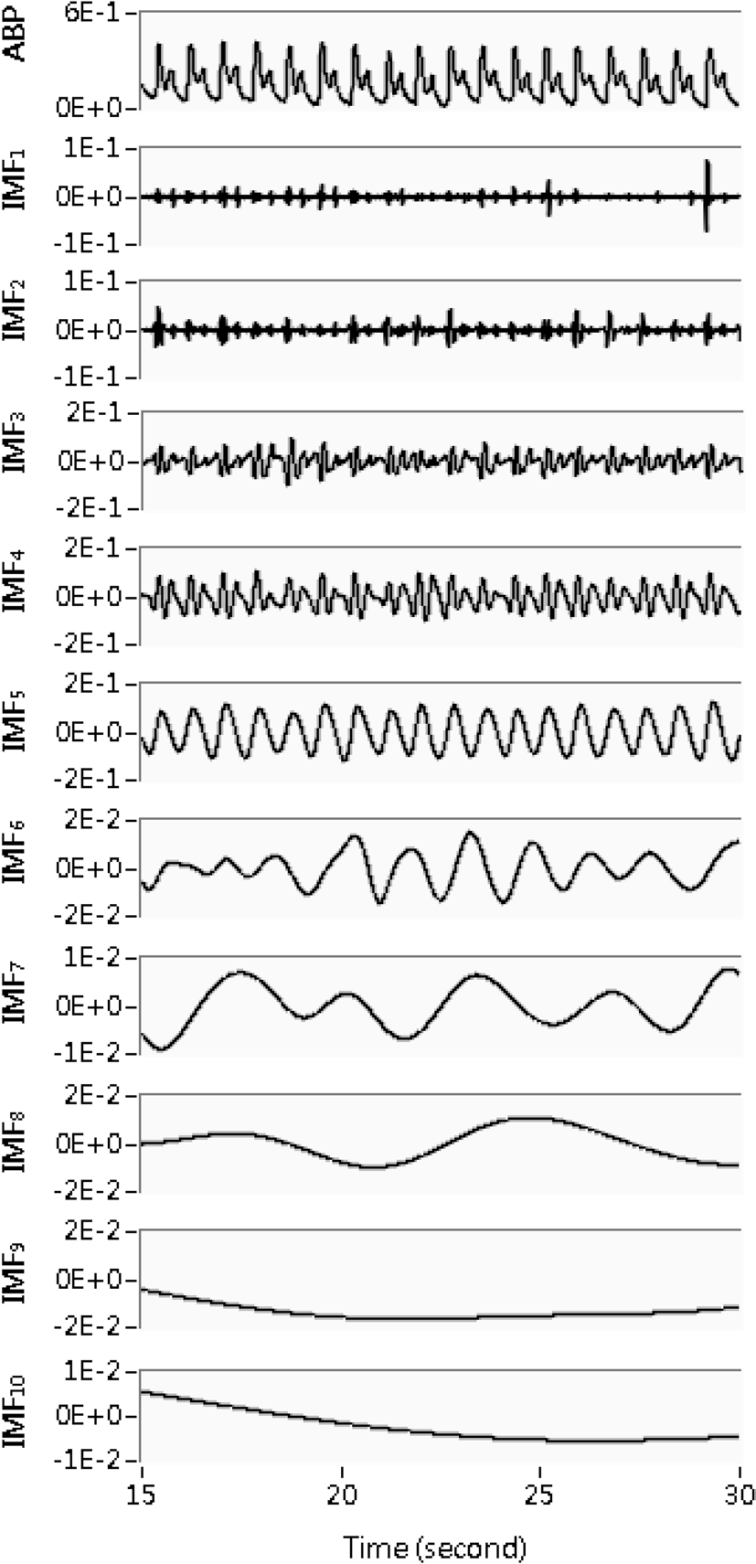
ABP signal and its ten corresponding IMFs

In this study, ABP signals were downsampled from 1000 to 200 Hz and subsequently decomposed into 10 IMFs by using CEEMD and a sifting process. Figure 4 shows one of the ABP signals and corresponding 10 IMFs. On the basis of the frequency bands of the IMFs [16–18], IMF_5_ and IMF_7_ were selected as the pulse beat and respiratory oscillation components, respectively.

### Computation of instantaneous frequency and period using the NDQ method

The NDQ method was adopted to compute the IF_resp_ and instantaneous period of pulse beat components. The steps of the NDQ method are as follows [15]:

- 1) Detect local maxima values of the absolute IMF.
- 2) Generate the upper envelope using a cubic spline.
- 3) Divide the IMF by the upper envelope to compute the normalized component *y*(*t*).
- 4) Repeat steps 1 to 4 until *y*(*t*) is equal to or less than unity, at which point *y*(*t*) is identified as the frequency-modulated *F*(*t*).
- 5) Compute instantaneous phase *ϕ*(*t*) using the following equation (7):
  7 $$\phi(t)=\arctan\,\frac{{\sqrt{1-{F^2}t}}}{F(t)}$$
- 6) Evaluate instantaneous frequency using the derivative of*ϕ*(*t*) and calculate the instantaneous period using the inverse instantaneous frequency.

### Instantaneous PRV

The instantaneous period of pulse beat was treated as the instantaneous pulse rate. To increase our sample size, the instantaneous pulse rate and IF_resp_ of 6-min time series were both segmented into a 5-min window size with a sliding increment of 10 s [43]. The frequency domain of iPRV was evaluated by examining each segment of the instantaneous pulse rate using the auto-power-spectrum method; LF PRV (0.04‒015 Hz; SNS and PNS activities), HF PRV (0.15‒0.4 Hz; PNS activity), LF/HF PRV ratio (balance between SNS and PNS), and VHF PRV (0.4‒0.9 Hz; peripheral regulation) were examined [15, 16]. To reduce the physiological difference between participants, LF, HF, and VHF PRV values were normalized as nLF, nHF, and nVHF PRV values, respectively.

### Determining the selection rates of features and the classification accuracy of the XCSR

**Fig. 5:**
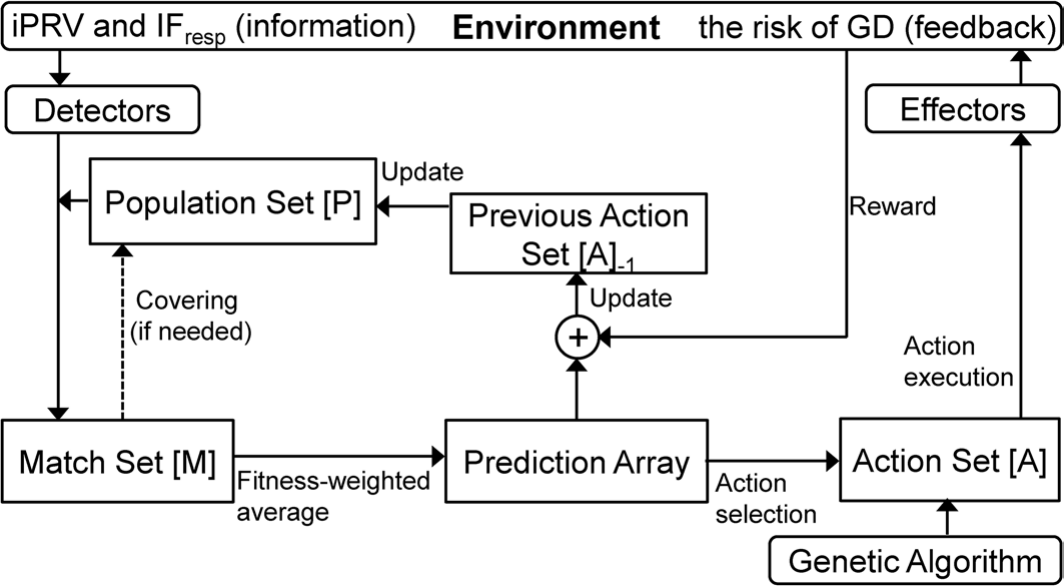
Overview of an XCSR (modified [24, 25])

LF PRV, HF PRV, VHF PRV, LF/HF ratio, nLF PRV, nHF PRV, nVHF PRV, and average of IF_resp_ were input as features into the XCSR for classification accuracy. The selection rate of each feature was also computed in the XCSR. The XCSR is a rule-based learning classifier system in which every classifier contains the following: a condition, which is encoded as interval_*e*_ = (*c*_***e***_, *s*_*e*_), *e* = 1…*d*, where *c*_***e***_, *s*_*e*_, and *d* are the center value, spread value, and dimension of attributes, respectively; an action, which is a reaction of the XCSR to the environment; a prediction, which is the expected reward; a prediction error, which is the error of the aforementioned prediction; and a fitness parameter, which is the quality of the classifier. The iterative process of the XCSR is as follows [24, 25] (Fig. 5).

- 1) The data from the environment are input into the XCSR, which then checks the condition of each classifier in the population [P] that contains the rule set. If the range of [*c*_*e*_ − *s*_*e*_, *c*_*e*_ + *s*_*e*_] of the condition matches the input data *z*_*e*_, all the matched classifiers are placed into the match set [M]. If no matched classifiers exist in [M], the covering operator of the XCSR generates a new classifier that covers the input data. The new classifier is input into [P]. The XCSR then searches the matched classifiers in [P] again.
- 2) The classifiers with the same action in [M] are used to calculate the fitness-weighted average of predictions. An action is then selected using the roulette-wheel method or maximum predicted value. All classifiers with the selected action are input into the action set [A]. The effector of XCSR outputs the selected action into the environment and receives a reward from the environment.
- 3) On the basis of this reward, the prediction, prediction error, and fitness values in [A] are updated using the reinforcement learning operation.
- 4) The XCSR also updates the classifiers in [P] per the classifiers in [A]. Furthermore, the genetic algorithm is executesd on [A] to generate better classifiers.

In the present study, the parameter setup of the XCSR was based on those used in previous empirical studies [24, 25, 44]. Population size was set to 400, and the number of rewards was set to 1000 and 0 for HGD and LGD, respectively. The classification accuracy of the XCSR was examined using the moving average per 50 exploitations, and the XCSR procedure was repeated 30 times to calculate the average accuracy. During these 30 repetitions, all classifiers in [P] were investigated. If the numerosity of the classifier (i.e., the number of copies of the same classifier) was higher than 2 and the prediction value of the classifier was 1000, this classifier was collected. Next, if the ranges of [*c*_*e*_ − *s*_*e*_, *c*_*e*_ + *s*_*e*_] of the conditions for these collected classifiers did not match the input features (LF PRV, HF PRV, VHF PRV, LF/HF PRV ratio, nLF PRV, nHF PRV, nVHF PRV, and average of IF_resp_), these conditions were ignored [24, 25]. The selection rate of each feature was determined by dividing the total number of the collected classifiers by the number of conditions that were not ignored.

### Statistical analysis

The Mann–Whitney *U* test was adopted to test for significant differences between participants with LGD and HGD in terms of their age and CIAS, IGDQ, SAM, and DEQ scores. To compare the difference between the baseline and stimulus results of participants with LGD and HGD, factorial analysis of variance (factorial ANOVA) was performed using time (the first, second, third, fourth, fifth, sixth, and seventh segments) as a within-subjects factor and state (baseline and stimulus) as a between-subjects factor. Factorial ANOVA was also used to test for differences between participants with HGD and LGD at baseline and during the stimulus experiment, with time (the first, second, third, fourth, fifth, sixth, and seventh segments) being used as a within-subjects factor and grouping (HGD and LGD) as a between-subjects factor. SPSS (version 22; IBM, Armonk, NY, USA) was used to perform the statistical analyses, and statistical significance was set at *p* < 0.01.

## Data Availability

The datasets used and/or analyzed during the current study are available from the corresponding author on reasonable request.

## Abbreviations

ABP: Arterial blood pressure
CEEMD: Complementary ensemble EMD
CIAS: Chen Internet addiction scale
EDQ: Discretely emotional questionnaire
EMD: Empirical mode decomposition
GD: Gaming disorder
HF: High frequency
HGD: High-risk GD
IF_resp_: Instantaneously respiratory frequency
IP: Instantaneous period
IGDQ: Internet GD questionnaire
IMF: Intrinsic mode function
iPRV: Instantaneous PRV
LF: Low frequency
LGD: Low-risk GD
nHF: Normalized HF
nLF: Normalized LF
nVHF: Normalized VHF
NDQ: Normalized direct quadrature
PNS: Parasympathetic nervous system
PRV: Pulse rate variability
SAM: Self-assessment manikin
SNS: Sympathetic nervous system
VHF: Very high frequency
XCSR: Extended classifier system with continuous real-coded variables
